# Bizarreness of Lucid and Non-lucid Dream: Effects of Metacognition

**DOI:** 10.3389/fpsyg.2019.02946

**Published:** 2020-01-09

**Authors:** Chunyun Yu, Heyong Shen

**Affiliations:** Center for Studies of Psychological Application, School of Psychology, South China Normal University, Guangzhou, China

**Keywords:** lucid dream, bizarreness, self-reflection and insight, prevalence, self-consciousness, continuity hypothesis

## Abstract

Dreams are usually characterized by primary consciousness, bizarreness and cognitive deficits, lacking metacognition. However, lucid dreaming (LD) is a type of consciousness state during which the dreamer is aware of the fact that he or she is dreaming, without leaving the sleeping state. Brain research has found that LD shares some common neural mechanisms with metacognition such as self-reflection. With a different metacognition level, the bizarreness of LD would also change. However, the difference in bizarreness between LD and non-LD was seldom explored, and individual differences were often neglected. In the present study, considering LD prevalence in Asia was rarely studied and related results in China and Japan were very different from each other, we first investigated the LD frequency of China in a standardized way. On that basis, we collected dreams of subjects who had relatively higher LD frequency and compared bizarreness density (BD) of LD and non-LD. Moreover, to explore the relationships of metacognition traits and BD, we also measured self-reflection and insight trait by Self-Reflection and Insight Scale. We found that 81.3% of subjects have experienced LD once or more, which is similar to findings in some western countries. Besides, BD was significantly lower in LD than in non-LD. Self-reflection and insight were inversely associated with dream bizarreness. These findings indicate that self-consciousness traits extend from waking to LD and non-LD state. As a particular consciousness state, LD may shed light on the research of consciousness and dream continuity. Future research on dream bizarreness is suggested to take dream types and metacognition differences into consideration.

## Introduction

In ancient China, Zhuang Zhou’s dream of becoming a butterfly was a famous story. In his dream, Zhuang Zhou turned into a butterfly, forgetting that he was a human being. When he woke up, he realized that he was still a human and began to think about whether he was a butterfly which was dreaming, just dreaming of becoming a person (Fang, 2010). From a psychological point of view, Zhuang Zhou lost normal self-reflection and insight function when dreaming. When he woke up, he regained these functions and could reflect on whether he was dreaming at that moment. The Dream Argument of Descartes also believed that individuals could not test whether they are dreaming or not in dreams (Haldane and Rosswrited, 1973).

Edelman (1992) proposed two kinds of consciousness: Primary consciousness is a simple consciousness shared by humans and mammals, including perception and emotion. Whereas secondary consciousness or higher-order consciousness enables a cognitive subject to think abstractly, recognize his or her own behaviors or emotions, and have the concept of past and future. In different states, the intensity and characteristics of human consciousness will change. When awake, individuals can have secondary consciousness to supplement primary consciousness. However, dreaming is mainly characterized by primary consciousness, lacking secondary consciousness (Hobson, 2009). In non-lucid dreams, metacognition like self-reflection was also found to be lower than in waking (Rechtschaffen, 1978; Bradley et al., 1992; Kahan and LaBerge, 1996).

However, there is sometimes an exception called lucid dreaming (LD). It is a kind of consciousness state during which the dreamer is aware of the fact that he or she is dreaming, without leaving the sleeping state (LaBerge and Rheingold, 1990). In that state, individuals may restore some reflective consciousness and sometimes have partial control over the content of their dreams (Dresler et al., 2014). LaBerge et al. (1981) and Fenwick et al. (1984) provided evidence for LD by letting participants demonstrate their lucid state during dream periods using predefined eye-movement signals. It was also found by Erlacher and Schredl (2010) that rehearsing in LD can enhance related performance in waking life. As a psychological phenomenon with a physiological basis, the objectivity of LD has been proved. This special state of consciousness is of considerable significance to the investigation of consciousness.

Many cognitive defects in dreams also occur in mental disorders. Freud (1958) said that psychotic episodes have something in common with dream characteristics. Jung (1934) also mentioned that if a person gets out of the bed and shows the content of the dream during dreaming, we can see some symptoms of dementia praecox in him. In dreaming, the prefrontal cortex of the brain is often inhibited, and cognitive functions like attention and working memory that normally work in waking state are offline (Fuster, 2015). Hobson (1997) suggested that dreaming can be seen as a model for psychosis. The lack of self-reflection in dreams is like the lack of insight in mental patients. The aminergic inhibition and cholinergic excitation shift the chemical balance within the brain, which is responsible for the delirium in dreams (Hobson et al., 2000).

In contrast, LD is very special, and it still retains higher-order consciousness functions including metacognition compared with non-LD. Blagrove (2011) proposed that whether dreams and waking life are continuous needs to be augmented by an insight dimension. It was proved that personal insight would increase by examining dream content (Edwards et al., 2015; Blagrove et al., 2019). In fact, there is also a continuity of insight function between wake and dream state. Neuroimaging and electroencephalogram (EEG) research showed that the frontal areas of the brain, which are related to psychotic insight, are highly activated in LD (Dresler et al., 2015; Voss et al., 2018). To some extent, LD can be seen as a model for insight into the psychotic state. It was found that metacognition is not completely deficient during both LD and non-LD (Kahan and LaBerge, 1994, 1996). In the special sleep state of LD, individuals can still have many cognition functions which usually appear when awake.

However, people generally have a tendency to think that there are many bizarre elements in LD contents. It is relevant to the low frequency of LD, and the fact there are many strange sleep phenomena often associated with LD, such as false awakening and out-of-body experience (Green and McCreery, 1994; Blanke and Arzy, 2005). Therefore, there is a discrepancy of LD bizarreness between brain research and common sense. It seems that lucid dreams are unrelated to schizotypy and dissociation (Knox and Lynn, 2014; Aviram and Soffer-Dudek, 2018), or at least, are related to them less strongly than other unusual sleep experiences (Watson, 2001). According to dream research, bizarreness is a very important feature of dream content and can be seen as a result of impaired cognitive processing (e.g., Hobson et al., 1987). With a high level of metacognition function, the bizarreness of LD content should be lower than that of non-LD content. Research on LD content needs to be carried out.

Although most people experience LD very occasionally, still, many people experienced LD at least once. The prevalence of LD was very different and controversial. In the only study on the prevalence of LD in China, 92% prevalence of LD was measured (Yu, 2008). It is the highest data in the world as far as we know. In another Asian sample, Japan, 47% LD prevalence was detected (Erlacher et al., 2008). The results of the two Asian samples are very different from each other. Ribeiro et al. (2016) suggested that methodological differences may lead to differences in the prevalence of LD. Hence, we measured the LD prevalence of China in a standardized way proposed by Schredl and Erlacher (2004).

There are not only differences in the level of metacognition between dream and waking, but also between dream and dream (Kahn and Hobson, 2005). Defining dreams as an intrinsically bizarre thing or accurate response to waking experiences does not explain the diversity of dreams, so the attention should be focused on the differences between dream experiences (review see Rosen, 2018). If the level of metacognition affects the bizarreness of dream, there will be more metacognitive activities and less bizarre elements in LD compared with non-LD. However, the difference in bizarreness between LD and non-LD was seldom explored, and individual differences were often neglected.

Therefore, this study first adopted a within-subject design to explore the dream bizarreness difference between LD and non-LD of the same individual. After that, we further explored how bizarreness differs on different levels of metacognitive traits. In the neuroimaging research of Dresler et al. (2012) about LD, increased activations were found in the right dorsolateral prefrontal cortex which is associated with self-focused metacognitive evaluation, and in the bilateral frontopolar areas which are implicated in the processing of one’s own thoughts and feelings. Filevich et al. (2015) also found that LD shares some common neural mechanisms in the frontopolar cortex and hippocampus with thought monitoring and self-reflection. Secondary consciousness like self-awareness is a key to LD, and its level may also affect the bizarreness of dreams. Thus, self-reflection and insight traits were chosen as metacognitive variables.

One thorny problem is that even for those who have a higher frequency of LD, this frequency is still not high. It is difficult to collect lucid dreams from different individuals through dream diaries or laboratory experiments. Induction techniques such as bedtime cues can greatly increase the chances of having LD (Schädlich and Erlacher, 2012), but this will reduce the ecological validity of the study. Taking these into account, we selected the most recent dream paradigm adopted by Domhoff (1996) to allow participants to report a lucid dream that has already taken place. Another problem to be noticed is that some studies measured dream bizarreness through a self-assessment method. For one thing, some individuals would tend to regard the strangeness of LD-related phenomena as the bizarreness of LD contents. It may lead to the overestimation of the LD bizarreness. For another, judgment criteria are different among subjects and cannot be objectively compared. Therefore, our present study used an other-rating method to reduce the error.

We hypothesized that (1) the bizarreness of non-LD is higher than that of LD for the same individual. (2) High metacognitive traits or self-consciousness of waking are related to the reduction of bizarreness in dreams.

## Materials and Methods

### Participants

Participants were all undergraduates or postgraduates of universities recruited from online student groups in Guangzhou. Overall, 326 persons (232 women, 94 men) completed our first survey. The mean age of the sample was 22.24 ± 2.96 years, from 18 to 33. According to the result of the first survey, 176 (54.0%) of these participants had a relatively higher LD frequency (equal or higher than 2–4 times a year). They were invited to take part in the next experiment which requires subjects to report dreams. Totally, 67 (38.1%) persons (51 women, 16 men) completed the second part, with an average age of 21.63 ± 2.92 years, from 18 to 29.

### Materials

The lucid dream frequency scale (Schredl and Erlacher, 2004) is composed of an eight-point rating question (0, never; 1, less than once a year; 2, about once a year; 3, about two to four times a year; 4, about once a month; 5, about two or three times a month; 6, about once a week; 7, several times a week) and a standard definition of LD (“*During LD, one is–while dreaming–aware of the fact that he or she is dreaming. It is possible to wake up deliberately, control the dream action, or observe the course of the dream with this awareness passively*”). The retest reliability of the scale was *r* = 0.89 in the student sample (Stumbrys et al., 2013). Moreover, we provided an example of lucid dream narrative as proposed by Saunders et al. (2016) to increase the confidence of results. An example in the study of Neider et al. (2011) was given. The dream recall frequency scale was a seven-point rating question (0, never; 1, less than once a month; 2, about once a month; 3, twice or three times a month; 4, about once a week; 5, several times a week; 6, almost every morning) with the retest reliability of *r* = 0.83 (Schredl et al., 2002). Results obtained can be recoded to get units in frequency per month or week by using the class means. These two questions were translated into Chinese.

Dream Collection adopted the recent dream paradigm (Domhoff, 1996), participates were asked to report a most recent lucid dream and a most recent non-lucid dream. They needed to describe these two dreams as fully and precisely as they could, including settings, people, animals, and so on. According to the classic method of Hall and Van de Castle (1966), 50–300 words of each report was required to judge. These were all presented in the online form, which is a valuable source of information that can provide enough privacy for participants to report more real and complete dreams (Voss et al., 2013).

Self-reflection and Insight Scale (SRIS: Grant et al., 2002) is the tool we used to measure metacognition traits of waking life during waking. It consists of Self-Reflection (SR) subscale and Insight (IN) subscale. SR refers to an understanding of one’s thoughts, feelings, and behavior, including 12 items. IN refers to cognition of one’s internal state, including 8 items. Liu et al. (2018) have translated it into Chinese version. The retest reliability of the two subscales is *r* = 0.81 and *r* = 0.61, respectively. In our study, the internal consistency reliability of SR (α = 0.82) and IN (α = 0.71) was also checked.

### Procedure

In order to provide sufficient privacy to ensure authenticity, we recruited participants in the college network communities of Guangzhou. Participants were told that this was a study of dreaming. By clicking on the web link, they completed a questionnaire including questions about the frequency of LD and dream recall. At the end of the questionnaire, they were invited to leave an email address if they were willing to participate in the follow-up study.

Based on the results of the LD frequency investigation, we screened the subjects for the second part. Among these 326 participants who completed the questionnaire, 176 people had a relatively higher LD frequency (equal or higher than 2–4 times a year). Of them, 149 subjects also left their mailboxes. Then, they were invited to participate in the second part including dream reports. Subjects needed to report a most recent lucid dream and a most recent non-lucid dream. After that, they finished the Self-Reflection and Insight Scale (SRIS). Altogether 67 subjects completed the dream report and submitted the spreadsheet as requested. Finally, we got 67 pairs of dreams. The first part was voluntary, while subjects who finished the second part would get feedback of their dreams and monetary compensation.

### Dream Bizarreness Scoring

The system of Revonsuo and Salmivalli (1995) was used to score the bizarreness of dream contents. This method consists of two steps. The first step is to identify 14 different kinds of elements in the dream report, including events, actions, place, time, animals, cognition, body parts, plants, objects, self, language, emotions, persons, and sensory experiences. Each element can only be categorized as one of the 14 elements. The second step is to score each element as either bizarre or non-bizarre. There are two kinds of possible bizarre elements: vagueness element and incongruous element. And the latter includes exotic elements, internally distorted or contextually incongruous elements, and impossible elements.

Two judges who were blind to the study purpose were trained together at first. Then the dream contents were scored independently by them. After that, judges crosschecked the scores and resolved disagreements by discussing together. In total, 110 (5.4%) of the 2032 elements were dropped because no agreement on the scores could be reached between the two judges. Of the final 1922 scored elements, 1813 elements were initially independently classified as the same content category; 1771 elements were initially independently scored as the same bizarreness category. Thus, the content agreement was 94.3%, and the bizarreness agreement was 92.1%. Bizarreness density (BD) was calculated in order to balance the difference in the word count of dream reports by dividing the number of bizarreness elements by the total number of elements.

### Data Analysis

Statistical analyses were applied by the IBM SPSS 19.0 software package for Windows. We used Spearman rank correlation to assess the relationship between LD frequency and non-LD recall frequency. ANOVA was applied to check the bizarreness difference between LD reports and non-LD reports within-subjects controlling for gender. Linear regression analysis was the statistical tool we used to assess the relationships of self-reflection and insight with bizarreness, respectively.

## Results

To analyze the prevalence of LD in China, we measured the frequency of LD. In total, 81.3% of 326 participants reported having at least one lucid dream in their life. According to the definition of Snyder and Gackenbach (1988), 30.4% of these subjects were frequent lucid dreamers (frequency equal or higher than once a month), and the other subjects were infrequent lucid dreamers. These are close to the data of the university student sample in German (see Table 1). The average frequency of LD was 1.02 ± 2.57 lucid dreams a month. The mean dream recall frequency was equal to 3.84 ± 2.30 times per week. Dream recall frequency and LD frequency were significantly related (*r* = 0.265, *p* < 0.0001).

**TABLE 1:** Lucid dreaming frequency of the Chinese and German sample.

**Categories**	**Relative frequency**	
	**Chinese present study (*N* = 326)**	**German Schredl and Erlacher (2004) (*N* = 439)**
Never	18.7%	18.0%
Less than once a year	12.0%	7.5%
About once a year	15.3%	10.9%
About 2 to 4 times a year	23.6%	26.7%
About once a month	11.7%	16.2%
About 2 to 3 times a month	11.7%	10.3%
About once a week	5.2%	8.0%
Several times a week	1.8%	2.5%

Bizarreness density values of LD reports were significantly lower than BD values of non-LD reports [*n* = 67, non-LD versus LD on BD: *F*(1,65) = 7.562, *p* = 0.008, mean ± SD LD: 0.15 ± 0.08 non-LD: 0.20 ± 0.11]. There was no significant main effect of gender [*F*(1,65) = 2.977, n.s.]. The interaction of dream type and gender was not significant [*F*(1,65) = 1.446, n.s.].

The mean self-reflection values of the 67 participants was 56.82 ± 6.62. There was a significant linear correlation of self-reflection and dream type with BD [*F*(2,131) = 10.689, *p* < 0.001, *R*^2^ = 0.14, *n* = 67]. Self-reflection was negatively correlated with BD (*B* = −0.004, *t* = −3.165, *p* = 0.002) (see Table 2). The mean insight values of the 67 subjects was 33.01 ± 4.26. There was a significant linear correlation of self-reflection and dream type with BD [*F*(2,131) = 9.914, *p* < 0.001, *R*^2^ = 0.13, *n* = 67]. Insight was inversely correlated with BD (*B* = −0.006, *t* = −2.929, *p* = 0.004) (see Table 3).

**TABLE 2:** Regression coefficients of dream type and self-reflection on bizarreness density.

					**95.0% Confidence**	
					**interval for B**	
					**Lower**	**Upper**
**Variable**	***B***	***SE***	**β**	***t* value**	**bound**	**bound**
Intercept	0.470	0.073	N/A	6.453	0.326	0.615
Dream type	−0.053	0.016	−0.273^∗∗^	−3.371^∗∗^	−0.085	−0.022
Self-Reflection	−0.004	0.001	−0.256^∗∗^	−3.165^∗∗^	−0.006	−0.001

*^∗∗^*p* < 0.01.*

**TABLE 3:** Regression coefficients of dream type and insight on bizarreness density.

					**95.0% Confidence**	
					**Interval for B**	
					**Lower**	**Upper**
**Variable**	***B***	***SE***	**β**	***t* value**	**bound**	**bound**
Intercept	0.436	0.067	N/A	6.496	0.303	0.569
Dream type	−0.053	0.016	−0.273^∗∗^	−3.354^∗∗^	−0.085	−0.022
Insight	−0.006	0.002	−0.238^∗∗^	−2.929^∗∗^	−0.009	−0.002

*^∗∗^*p* < 0.01.*

## Discussion

### Lucid Dreaming Prevalence in the Chinese Sample

In the present study, we first investigated the prevalence and frequency of LD in China. Our results showed that the prevalence of LD in Chinese university students sample is 81.3%, which is very similar to what was found in some other countries: In the German sample, 82% of the participants reported the occurrence of at least one lucid dream (Schredl and Erlacher, 2004); among Israeli students, 78.61% of LD prevalence was found by using a clear definition (Aviram and Soffer-Dudek, 2018); likewise, 77.2% of the Brazilian subjects had at least one LD experience in their whole lifetime (Mota-Rolim et al., 2013); when the definition of LD was presented in French students, the prevalence found was 81.05% (Ribeiro et al., 2016). The previous study in China found a high LD prevalence of 92%. However, only a short question about LD frequency was asked in that study. The lack of a clear definition and an example could cause subjects to overestimate LD frequency and prevalence (Snyder and Gackenbach, 1988). Our research gave a clear definition and an example of LD. Different descriptions of LD may result in our different findings. In Japan, another Asian country, the incidence rate of LD measured was only 47%. Japan has a different history of the understanding on dreams from the West. Even if dreams are considered as a scientific phenomenon in the modern century, Japanese still retains animism on dreams since ancient time (Koyama, 1995). Thus, dreaming is very private for Japanese and hard to talk about, especially strange dreams like LD. The attitude of Japanese people toward dreams might cause the low prevalence of LD in previous studies. In short, our present study found that as a common physiological phenomenon of humans, the LD prevalence in China is not much different from that in Western countries.

### Relationships of Dream Type and Metacognition Traits With Dream Bizarreness

The bizarreness of LD and non-LD were checked within-subjects. We found that the BD of LD reports was significantly lower than that of non-LD reports. As mentioned before, bizarreness can be seen as a result of metacognition reduction in dreams. Our results on dream contents showed that the decrease of metacognition activity is not so prominent in LD. That is consistent with the existing brain science research mentioned before that LD has some common neural mechanisms with thought monitoring and self-reflection. Watson (2001) found that LD was weakly correlated with schizotypy and dissociation, which are often seen as bizarre cognition phenomena. However, Aviram and Soffer-Dudek (2018) proposed that associations of symptoms with LD may due to the use of LD induction techniques which cause disturbed sleep, instead of LD *per se*. Gackenbach (1988) measured the bizarreness of LD and non-LD on 21 elements, whereas the bizarreness scores of non-LD were only significantly higher than that of LD on 3 kinds of elements. In our study, we used a within-subject design to balance the effects of individual differences. It may explain the difference between our results.

Ogilvie et al. (1982) thought that individuals begin to realize the dream state in LD is because they have noticed the bizarre things. However, there are also many strange elements in non-LD, and individuals cannot recognize them. Hobson (2009) believed that bizarreness or inconsistency is often unable to be recognized in the dream state. Therefore, there is no necessary relationship between having LD and noticing bizarre elements. Another possible explanation is suggested by our result: For an individual, the higher level of metacognition in LD may allow him or her to realize the sleeping state. In other words, dream bizarreness may not be the cause of LD emergence, but the result of metacognition level changes followed by LD state.

Furthermore, we also explored the relationships between metacognition traits and dream bizarreness. Self-reflection and insight were inversely associated with dream bizarreness. Our results suggested that metacognition traits are not only reflected in waking, but also in dreaming; not only reflected in non-LD, but also in LD. Metacognitive activity is often inhibited in dreams, but this kind of reduction is also different among different individuals. Individuals with higher metacognition traits would still have more metacognition activities in dreams and lower dream bizarreness values.

### Continuous Self-Consciousness Across LD, Non-LD, and Waking

There has always been a controversy between continuity and discontinuity in the field of dream research. The continuity hypothesis is based on overlaps of dream and wakefulness (e.g., Schredl and Hoffman, 2003; Malinowski and Josie, 2015). Sometimes what people have in their dreams are also reflected in their waking life. In contrast, the discontinuity hypothesis focuses on the differences between waking and dreaming (eg., Jung, 1960; Hobson et al., 1987). Although the metacognition function in LD we found is far better preserved than in non-LD, it is still incomplete compared with the waking state. Most lucid dreams are not that lucid in general. A study of frontal areas in LD state demonstrated that LD is more likely a middle state between waking and non-LD (Voss et al., 2009). Individuals in LD still cannot fully have memories of the past and future or maintain the awareness of their own state. Nevertheless, the interruption of self-consciousness is not only a characteristic of dream cognition but also a characteristic of waking cognition (Horton, 2017). For example, a person may mistake the date of the previous day for today when he or she is awake. There are many things in common between waking and dreaming.

Partly due to the memory consolidation process in the sleeping state, contents of dreaming are not continuous with waking-life experiences (Horton and Malinowski, 2015). Nevertheless, Stumbrys (2011) proposed that the lucidity in dreams, like mindfulness in wakefulness, presents a possible continuity in the self-consciousness across the sleep-wake cycle. The results of our study confirmed this opinion. We found that higher self-reflection and insight traits are related to less dream bizarreness. Participants showed consistency in LD, non-LD, and waking, which supported the continuity hypothesis of waking and dreaming. Self-reflection and insight traits extend from waking life to dreams.

### Limitation and Suggestion

First of all, participants were all college students in the present study. The university student sample was used by most of the existing research on LD prevalence. However, there are age differences in the prevalence and frequency of LD (Voss et al., 2012). Future research could expand the group of participants and take demographic information into account. Mota et al. (2016) found that psychotic patients who have LD experience didn’t present milder psychiatric symptoms than patients who don’t have LD experience. LD may have very different meanings for normal people and psychiatric patients. Therefore, psychiatric patients should also be taken into consideration in the future.

Secondly, the present study found that LD prevalence in China is very similar to that in some western countries. However, it’s not enough to assert that culture has less relevance to the prevalence of LD. Individual differences like mindfulness, which is associated with the LD prevalence, are sometimes strongly different in different cultures (Stumbrys et al., 2015). LD prevalence may also vary within a culture at different times. Therefore, in order to explore the degree of the relationship between LD prevalence and culture, LD prevalence across cultures and at different times within a culture are planned to be investigated. Aviram and Soffer-Dudek (2018) mentioned that assessing LD with a single item would overlook the complexity of LD. Thus, LD characteristics like awareness and control should also be investigated separately.

Thirdly, the two judges, although not knowing the research hypothesis, could easily tell whether a dream was lucid or not from its content. Although the scoring had objective criteria and there were two independent judges to improve reliability, it would be meaningful to explore the influence of knowing lucidity on dream bizarreness scoring in the future. In this study, we only preliminarily explored the bizarreness difference between LD and non-LD. In order to explore more detailed changes in the bizarreness of LD, future research may divide LD into different stages (e.g., dream prior to lucidity, dream during lucidity), which are judged separately for bizarreness.

What’s more, it is hard to collect a complete lucid dream from each subject since LD seldom occurs. Besides, LD is particularly susceptible to suggestion before sleep. Therefore, this study only collected already happened dreams, using the most recent dream paradigm to ask each participant to report a most recent LD. However, errors may still occur because of memory bias and the fact that only one lucid dream is collected from each person. In future research, it would be better to ask subjects to keep a long-term dream diary without telling them about LD to get enough lucid dreams from each subject. And then identify lucid dreams and analyze these dreams together.

In addition, this present study is correlational, so no causal inference can be made. Other metacognition aspects like reality monitoring are also closely related to LD (Corlett et al., 2014). Dopamine can mediate metacognitive activities, such as self-awareness and reality monitoring (Schnider et al., 2010; Joensson et al., 2015). On the premise of not harming the health of subjects, drugs that promote dopamine secretion can be used to see the changes of dream bizarreness in future studies.

## Conclusion

Considering LD prevalence was rarely studied and related results were controversial in Asian countries, we first investigated the LD frequency of China in a standardized way. We found that the prevalence of LD in China is similar to findings in western countries. To the best of our knowledge, our present study is the first study to compare dream bizarreness of LD and non-LD contents within subjects. We found that the bizarreness of LD is lower than that of non-LD, which also proved that LD is not as strange as usually considered. In general, dream bizarreness is related to individual differences in metacognition traits, and subjects with higher self-reflection and insight would have lower dream bizarreness.

The results of this study revealed that there is a kind of continuous self-consciousness across waking, LD, and non-LD state. As a special consciousness state, LD may shed light on the research of consciousness and dream continuity hypothesis. Based on our findings, future research is suggested to treat dream bizarreness in a more general way. Dream types, individual differences in metacognition should be taken into consideration.

## Data Availability Statement

The datasets generated for this study are available on request to the corresponding author.

## Ethics Statement

The studies involving human participants were reviewed and approved by the School of Psychology, South China Normal University, Human Research Ethics Committee. Written informed consent for participation was not required for this study in accordance with the national legislation and the institutional requirements.

## Author Contributions

CY designed this research and finished it. HS contributed to the manuscript submitting and provided the language help.

## Conflict of Interest

The authors declare that the research was conducted in the absence of any commercial or financial relationships that could be construed as a potential conflict of interest.
